# VEXAS syndrome in a female with constitutional monosomy X

**DOI:** 10.1016/j.ero.2025.10.002

**Published:** 2025-10-30

**Authors:** Nikolas Ruffer, Simon Melderis, Olaf Determann, Lana Harder, Isabell Haase, Ina Kötter, Anja Lüdemann, Martin Krusche

**Affiliations:** 1 Division of Rheumatology and Systemic Inflammatory Diseases, III. Department of Medicine, University Medical Center Hamburg-Eppendorf, Hamburg, Germany; 2 MVZ HPH Institut für Pathologie und Hämatopathologie, Hamburg, Germany; 3 Institut für Tumorgenetik Nord, Kiel, Germany; 4 MVZ Rheumatologie und Autoimmunmedizin, Hamburg, Germany

## Abstract

The VEXAS (vacuoles, E1 enzyme, X-linked, autoinflammatory, somatic) syndrome is an autoinflammatory disorder that is caused by an acquired deficiency of the *UBA1* gene in haematopoietic progenitor cells and predominantly affects elderly males. However, ultrarare cases of female VEXAS patients with acquired X chromosome monosomy in the bone marrow along with an additional mutation in *UBA1* have been reported. Herein, we report the case of 84-year-old female patie that developed the VEXAS syndrome in the context of constitutional monosomy X (Turner syndrome). The present case challenges the pervasive conception of the VEXAS syndrome as a disease confined to elderly males and informs the diagnostic considerations of clinicians.

Dear Editor,

The VEXAS (vacuoles, E1 enzyme, X-linked, autoinflammatory, somatic) syndrome is an autoinflammatory disorder that is caused by an acquired deficiency of the *UBA1* gene in haematopoietic progenitor cells. The striking male predominance in this X-linked disorder is reflected by current disease models, which propose the physiologic absence (in males) or loss of one X chromosome (functional or constitutional) along with an additional mutation in *UBA1* for clinical disease manifestation [1]. Exceptional reports of female VEXAS patients with constitutional X deletion or acquired X chromosome monosomy in the bone marrow and apparently similar VEXAS features in both sexes support this notion [1]. A recent systematic review [1] reported 9 published cases of female patients with VEXAS syndrome in the literature including 1 case of constitutional monosomy X, which highlights the need for additional data to delineate the clinical features of the VEXAS syndrome in women. In contrast to the available reports, the prevalence of the VEXAS syndrome has been estimated 1 in 26 238 women older than 50 years [2]. Whether female VEXAS patients differ in clinical presentation depending on the underlying disease mechanism is also uncertain. In this context, we report the ultrarare case of a female patient with the VEXAS syndrome and constitutional monosomy X.

A 84-year-old woman with a history of infertility developed an inflammatory syndrome (maximum C-reactive protein concentration, 192 mg/L) characterised by recurrent episodes of auricular chondritis, which resolved in response to glucocorticoid therapy. In addition, erythematous plaques involving the trunk, legs (Fig A), and arms, ocular inflammation, and bilateral pulmonary infiltrates resistant to diuretics and antibiotics (Fig B) were noted. The complete blood count demonstrated the presence of macrocytic anaemia (haemoglobin, 9.9 g/dL; mean corpuscular volume, 113 fL). Based on these findings, the VEXAS syndrome was suspected, and a pathogenic *UBA1* p.Met41Val variant (c.121A>C, variant allele frequency: 31%) was confirmed by Sanger sequencing 1 year after disease onset (Supplementary Table S1, Supplementary Table S2). Further evaluation of the bone marrow revealed the typical vacuolisation in myeloid precursors associated with the VEXAS syndrome (Fig C) and established the presence of a concomitant myelodysplastic syndrome (very low risk by Revised International Prognostic Scoring System). Cytogenetic analysis of the bone marrow disclosed the isolated loss of 1 X chromosome in 96% of the cells. Comparative evaluation by fluorescence in situ hybridisation analysis from the peripheral blood revealed X chromosomal mosaicism (including fractions with a female karyotype, predominant loss of 1 X chromosome and additional presence of an X chromosome) compatible with a diagnosis constitutional monosomy X (Turner syndrome) (Fig D). Subsequently, immunosuppressive therapy with methotrexate and prednisolone was introduced. However, the further disease course was characterised by recurrent flares and dependence on repetitive pulses of glucocorticoids to control disease activity. After failure of etanercept and filgotinib therapy, the patient was enrolled in a clinical trial.

**Figure:**
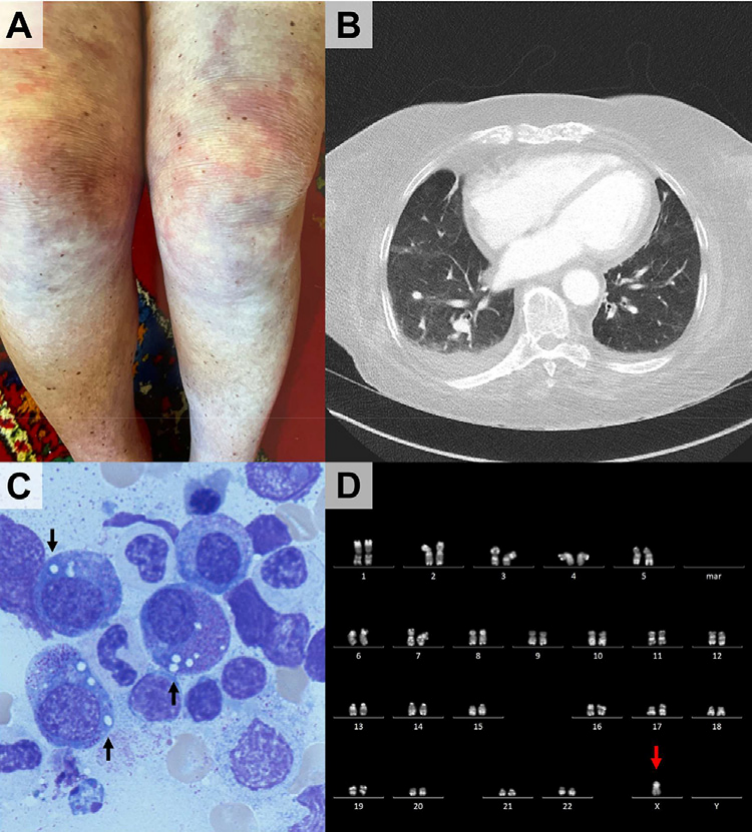
A, Multiple erythematous plaques localised at the thighs and knees. B, A computed tomography scan of the chest (axial plane) disclosed bilateral pulmonary infiltrates and pleural effusion. C, Diagnostic evaluation of the bone marrow aspirate (Pappenheim stain, ×1000) demonstrated a prominent vacuolisation of granulocytic precursors (black arrows). D, Peripheral blood karyotyping with R-banding revealed the previously unknown loss of one X chromosomecompatible constitutional monosomy X (red arrow).

The present case illustrates that the VEXAS syndrome can manifest in females in the context of constitutional monosomy X and informs the diagnostic considerations of clinicians. Genetic testing of our patient unveiled the underlying mechanism of disease manifestation and provided an explanation for the reported infertility.

Our patient clearly represents an ‘outlier’ that challenges the pervasive conception of the VEXAS syndrome as a disease confined to elderly males [3]. The diagnosis of the VEXAS syndrome should, therefore, be guided by clinical findings and must not be dismissed based on female sex or age. This perspective is reinforced by the recent report of a 23-year-old man with polychondritis, episcleritis, and macrocytic anaemia, which was ultimately diagnosed as VEXAS syndrome [4].

## Competing interests

The authors declare no competing financial interests.

## Appendix Supplementary materials

Image, application 1

## Footnotes

Supplementary material associated with this article can be found in the online version at doi:10.1016/j.ero.2025.10.002.
