# Supplementary material for: VEXAS syndrome in a female with constitutional monosomy X
Source: EULAR Rheumatol Open. 2025 Oct 30;1(4):359–60. doi: 10.1016/j.ero.2025.10.002 (PMC13292419; doi:10.1016/j.ero.2025.10.002)
Supplement: Supplementary file 1 [file mmc1.docx]

**Supplementary Table S1** Laboratory investigations and treatment prior to diagnosis of VEXAS syndrome.

|  | 12 months prior to VEXAS diagnosis | 11 months prior to VEXAS diagnosis | 3 months prior to VEXAS diagnosis | 1 month prior to VEXAS diagnosis | suspicion of the VEXAS syndrome |
| --- | --- | --- | --- | --- | --- |
| haemoglobin (g/dL) | 12.3 | 11.6 | 9.9 | 11.5 | 11.2 |
| MCV (fl) | 98 | 105 | 113 | 104 | 105 |
| MCH (pg) | 34.6 | 35 | 36 | 35.6 | 35.7 |
| MCHC (g/dL) | 35.1 | 33 | 32 | - | 93.9 |
| RDW | 12.8% | - | - | 15.6 | 17 |
| leukocyte count (/nL) | 5.8 | 7.0 | 6.6 | 4.5 | 5.2 |
| lymphocyte count (/nL) | 1.0 | 1.3 | 0.8 | 1.93 | 1.23 |
| Monocyte count (/nL) | 0.32 | 0.38 | 0.26 | 0.18 | 0.28 |
| platelet count (/nL) | 219 | 240 | 258 | 203 | 289 |
| C-reactive protein (mg/L) | 126.7 | 181.7 | 36.7 | - | 36.8 |
| ferritin (µg/L) | - | 757 | 232 | - |  |
| treatment | - | - | - |  | methotrexate + prednisolone |

**Supplementary Table S2** Disease features of the reported case.

| **Disease features** | **Presence** |
| --- | --- |
| fever of unknown origin | No |
| Auricular and/or nasal chondritis | Yes |
| Neurtrophilic dermatosis or urticaria-like lesions (Bx) | No |
| Leukocytoclastic vasculitis or leukocytoclasia | No |
| Non-infectious periorbital swelling | No |
| Recurrent, non-infectious inflammatory eye disease | Yes |
| Non-infectious ground glass or nodular pulmonary disease | Yes |
| Unprovoked or recurrent thromboembolic disease | No |
| Steroid dependency | Yes |
| Erythema nodosum | No |
| Recurrent urticaria / urticarial plaques | Yes |
| Injection site reaction to anakinra | No |
| Inflammatory arthritis | Yes |
| Vasculitis (any size), relapsing / recurrent or with lack of response to SOC | No |
| Pericarditis / Myocarditis | No |
| Exsudative pleural or pericardial effusion | Yes |
| Testicular inflammation | No |
| Sensorineural hearing loss | No |
| Atypical or opportunistic infection including nontuberculous mycobacterial infection | No |
| Nephrotic syndrome with renal amyloidosis | No |
| Interstitial nephritis | Yes |
| Vacuoles on myeloid or erythroid precursor cells on marrow aspirate | No |
| Macrocytosis or macrocytic anemia | Yes |
| MDS or myelodysplasia | Yes |
| Thrombocytopenia | No |
| Monocytopenia | No |
| Lymphopenia | No |
